# Phenolic Compounds from *Olea europaea* L. Possess Antioxidant Activity and Inhibit Carbohydrate Metabolizing Enzymes *In Vitro*

**DOI:** 10.1155/2015/684925

**Published:** 2015-10-08

**Authors:** Nadia Dekdouk, Nicola Malafronte, Daniela Russo, Immacolata Faraone, Nunziatina De Tommasi, Souad Ameddah, Lorella Severino, Luigi Milella

**Affiliations:** ^1^Facultè des Sciences de la Nature et de la Vie, Universitè des Freres Mentouri Constantine, route de Ain El Bey, 25 000 Constantine, Algeria; ^2^Dipartimento di Farmacia, Università di Salerno, Via Giovanni Paolo II 132, Fisciano, 84084 Salerno, Italy; ^3^Department of Science, Basilicata University, Viale dell'Ateneo Lucano 10, 85100 Potenza, Italy; ^4^Department of Veterinary Medicine and Animal Production, Division of Food and Veterinary Toxicology, University of Naples Federico II, Via Delpino 1, 80137 Naples, Italy

## Abstract

Phenolic composition and biological activities of fruit extracts from Italian and Algerian *Olea europaea* L. cultivars were studied. Total phenolic and tannin contents were quantified in the extracts. Moreover 14 different phenolic compounds were identified, and their profiles showed remarkable quantitative differences among analysed extracts. Moreover antioxidant and enzymatic inhibition activities were studied. Three complementary assays were used to measure their antioxidant activities and consequently Relative Antioxidant Capacity Index (RACI) was used to compare and easily describe obtained results. Results showed that *Chemlal*, between Algerian cultivars, and *Coratina*, among Italian ones, had the highest RACI values. On the other hand all extracts and the most abundant phenolics were tested for their efficiency to inhibit *α*-amylase and *α*-glucosidase enzymes. *Leccino*, among all analysed cultivars, and luteolin, among identified phenolic compounds, were found to be the best inhibitors of *α*-amylase and *α*-glucosidase enzymes. Results demonstrated that *Olea europaea* fruit extracts can represent an important natural source with high antioxidant potential and significant *α*-amylase and *α*-glucosidase inhibitory effects.

## 1. Introduction

*Olea europaea* L. is a typical fruit-tree widely cultivated in the Mediterranean area, belonging to Oleaceae family, even that its cultivation has been extended to many other regions of the world [1]. The olive fruit contains high concentrations of phenolic compounds that can range from 1 to 3% of the fresh pulp weight [2]. The main classes of phenols in olive fruit are phenolic acids, phenolic alcohols, flavonoids, and secoiridoids [3]. Phenolic acids, phenolic alcohols, and flavonoids occur in many fruits and vegetables belonging to various botanical families, whereas secoiridoids are exclusively present in Oleaceae family [3]. Phenolic acids in olive fruits include gallic,* p*-hydroxybenzoic, protocatechuic, vanillic, syringic, caffeic, ferulic,* p*-coumaric, and sinapic acids [4]. Tyrosol [(*p*-hydroxyphenyl)ethanol] and hydroxytyrosol [(3,4-dihydroxyphenyl)ethanol] are the most abundant phenolic alcohols in olive fruit [5]. The flavonoids include flavonol glycosides such as luteolin-7-glucoside and rutin as well as anthocyanins, cyanidin 3-*O*-glucoside, and cyanidin 3-*O*-rutinoside [6]. In some cultivars, delphinidin glycoside has been described [7]. Oleuropein and verbascoside are the principal secoiridoids present in olive fruit. The growing interest in olive polyphenols is due to the fact that they may play an important role in human health; in fact it is well known that the decreased incidence of cardiovascular diseases in the Mediterranean area has been partly attributed to the consumption of olive products [8]. Hyperglycemia is another important factor in cardiovascular damage, working through different mechanisms, like the reactive oxygen species (ROS) accumulation that, in turn, promote cellular damage and contribute to the diabetic complications development and progression [9]. The prevalence of diabetes mellitus and associated comorbidities has increased worldwide in recent decades. Therefore, the use of substances or agents that reduce postprandial hyperglycemic and oxidative stress may be therapeutic for diabetics [10, 11].

The aim of this work was to identify and quantify phenolic compounds in ripe fruits of two Algerian (*Chemlal* and* Sigoise*) and five Italian (*Coratina*,* Frantoio*,* Leccino*,* Maiatica*, and* Ogliarola*) cultivars of* Olea europaea* besides their total phenolic and total tannin contents. The total antioxidant activities of the ethyl acetate extracts were also carried out using 3 different assays. We have evaluated the extract and pure compound for *α*-amylase and *α*-glucosidase inhibitory activities. Results evidenced significant differences among investigated* Olea europaea* fruit extracts. Several studies reported the antidiabetic activity of* Olea europaea* leaves and oils [12], while, to the best of our knowledge, no investigation has been carried out on *α*-amylase and *α*-glucosidase inhibition of olive fruit extracts.

## 2. Materials and Methods

### 2.1. Chemicals and Apparatus

Folin-Ciocalteu reagent, sodium carbonate, bovine serum albumin (BSA), acetate buffer, sodium dodecyl sulphate (SDS), triethanolamine, ferric chloride, sodium acetate trihydrate, 2,4, 6-tripyridyl-s-triazine (TPTZ), 1,1-diphenyl-2-picrylhydrazyl (DPPH) radical, *β*-carotene, linoleic acid, Tween 20, sodium phosphate, sodium chloride, potassium sodium tartrate tetrahydrate, 3,5-dinitrosalicylic acid, sodium hydroxide, *α*-amylase, starch, *α*-glucosidase, potassium phosphate monobasic, 4-nitrophenyl *α*-D-glucopyranoside, and butyl hydroxytoluene (BHT) were acquired from Sigma-Aldrich, Milan (Italy). Standards as tannic acid, gallic acid, 6-hydroxy-2,5,7,8-tetramethylchroman-2-carboxylic acid (Trolox), and acarbose were purchased from Sigma-Aldrich, Milan (Italy). Solvents as* n*-hexane, ethyl acetate, acetonitrile, methanol, hydrochloric acid, chloroform, and glacial acetic acid were purchased from Carlo Erba (Milan, Italy). HPLC grade solvents, as methanol and Trifluoroacetic acid (TFA), were acquired from Romil (Cambridge, UK). All spectrophotometric measurements were done on a UV/Vis spectrophotometer (SPECTROstar^Nano^, BMG Labtech) and each reaction was performed in triplicate.

### 2.2. Sample Collection and Extraction Procedure

Fruits of seven different cultivars of* Olea europaea* were collected in Italy (*Coratina*,* Frantoio*,* Leccino*,* Maiatica*, and* Ogliarola* cultivars) and Algeria (*Sigoise* and* Chemlal* cultivars) in October 2012. Olive fruits were handpicked in the harvest season and were immediately frozen in liquid nitrogen to block the enzymatic activities. Healthy fruits (200 g), without any physical damage or kind of infection, were selected. A voucher specimen for each sample is deposited in the Herbarium of the Faculty of Natural and Life Sciences (Université des Frères Mentouri Constantine, Algeria). The samples were lyophilised and then extracted by maceration using ethyl acetate. It has been demonstrated that this solvent is better than others to extract phenolics [13]. Solvent has been replaced for 3 times and obtained extracts have been dried using a rotary evaporator. The dried ethyl acetate extracts were defatted by acetonitrile/*n*-hexane partition and the acetonitrile fractions were used for further analysis, because it has been previously reported that phenolic concentration is higher in the most polar fraction than in the less polar fraction [14].

### 2.3. Total Polyphenolic Content

Total polyphenolic content (TPC) was determined by using Folin-Ciocalteu reagent as previously described [15]. Diluted sample (75 *μ*L) was added to 425 *μ*L of distilled water, 500 *μ*L of Folin-Ciocalteu reagent, and 500 *μ*L of a sodium carbonate aqueous solution (10% w/v). The mixture was stirred and left in the dark for 60 min. Absorbance was measured at 723 nm and gallic acid was used as reference standard. Results were expressed as mg of gallic acid equivalents (GAE)/g of extract.

### 2.4. Tannin Content

In this study, protein (BSA) precipitation assay was applied to estimate* O. europaea* extract total tannin content [16]. The extracts were precipitated with BSA and after centrifugation the precipitates were dissolved in 1 mL of 1% SDS, 5% triethanolamine solution. Ferric chloride reagent (250 *μ*L) was added, and the solutions were mixed immediately. After 30 minutes, the absorbance was read at 510 nm and the results were expressed as mg of tannic acid equivalent (TAE)/g of extract.

### 2.5. Antioxidant Activity

#### 2.5.1. Radical-Scavenging Activity (2,2-Diphenyl-1-picrylhydrazyl, DPPH)

Radical-scavenging ability was determined by DPPH test as previously described [17]. For analysis, 300 *μ*L of diluted sample was added to 1200 *μ*L of DPPH solution (100 *μ*M). The ability of the extracts to scavenge the DPPH free radical was determined at 515 nm after 30 minutes of incubation, in the dark and at room temperature. Sample was replaced by methanol in the negative control, whereas Trolox was used as standard. Results were expressed as mg of Trolox equivalent (TE)/g of dried extract.

#### 2.5.2. Ferric Reducing Antioxidant Power (FRAP)

The Ferric Reducing Antioxidant Power of extracts was determined using FRAP assay [18]. Briefly, 150 *μ*L of appropriately diluted sample (150 *μ*L of methanol for the blank) was added to 1350 *μ*L of FRAP reagent and incubated at 37°C for 40 min in the dark. FRAP reagent was prepared fresh before experiment and it was prepared by mixing 300 mM acetate buffer in distilled water pH 3.6, 20 mM FeCl_3_ 6H_2_O in distilled water, and 10 mM TPTZ in 40 mM HCl in a proportion of 10 : 1 : 1. The reduction of a colorless ferric complex (Fe^3+^-tripyridyltriazine) to a blue-colored ferrous complex (Fe^2+^-tripyridyltriazine) by action of electron-donating antioxidants was determined at 593 nm. Trolox was used as standard and FRAP values were expressed as mg of Trolox equivalents (mg TE)/g of dried extract.

#### 2.5.3. Lipid Peroxidation Inhibition

The ability of extracts to prevent the inhibition of lipid peroxidation was carried out by *β*-carotene bleaching assay (BCB) [19]. A stock solution of *β*-carotene/linoleic acid was made by dissolving 0.2 mg of *β*-carotene in 0.2 mL of chloroform, linoleic acid (20 mg), and Tween 20 (200 mg). The chloroform was completely removed by rotary evaporator and distilled water (50 mL) was added with oxygen. The resulting emulsion was vigorously stirred. Aliquots (9.5 mL) of the mixture were transferred to test tubes containing 0.5 mL of sample (the final concentration for all tested samples was 0.1 mg/mL) or methanol as blank. BHT was used as a positive standard. The tubes were placed at 50°C for 3 h. The absorbance was monitored at 470 nm for 180 minutes and measured every 30′. Results were expressed as percentage of antioxidant activity (AA) measured on the basis of *β*-carotene bleaching inhibition and calculated as follows:

(1) AA%=1−Abs  sampleT0′−Abs  sampleT180′Abs  blankT0′−Abs  blankT180′×100.

### 2.6. HPLC-DAD Analysis

The extracts were analysed by reverse phase HPLC on an Agilent 1200 series (Agilent Technologies, Palo Alto, CA, USA) equipped with a binary pump (G-1312A), an autosampler (G-1329A), 1315-D Diode-Array Detector (DAD), and Onyx monolithic column (50 × 2 mm C18, Phenomenex, Italy). The mobile phase consisted of two solvents: acidified milli-Q water (Millipore, Bedford, MA, USA) with 0,1% TFA (A) and methanol (B), starting with 0% B and using a gradient to obtain 0% B at 1 min, 0–30% B at 6.7 min, 30–70% B at 13.70 min, 70–90% B at 14.50 min, and 90% B at 19.70 min. Samples were dissolved in water and 50 *μ*L of each sample was used for HPLC-DAD analysis. The flow rate was 0.6 mL/min and chromatograms were recorded at 278 nm.

The quantification of phenolic compounds was carried out using the same HPLC-DAD method applied for the analysis, with the respective standard. To assess the validity of the method, all test parameters were carefully chosen to cover the range of samples and concentrations involved. The linearity of standard curve was expressed in terms of the determination coefficient plots of the integrated peaks area* versus* concentration of the same standard and expressed as recovery (%) of phenols. These equations were obtained over a wide concentration range in accordance with the levels of these compounds in the samples. The system was linear in all cases (*r* > 0.99). Three replicates on the same day were carried out.

### 2.7. Antidiabetic Activity

#### 2.7.1. *α*-Amylase Inhibitory Activity

The inhibitory activity of *α*-amylase was assayed using 10 *μ*L of 20 mM sodium phosphate buffer (pH 6.9 with 6 mM NaCl) containing 0.5 mg/mL *α*-amylase (50 Units/mg) and then incubated at 25°C for 10 min with 10 *μ*L of extract. Extracts were dissolved in 10% methanol-buffer solution and tested at different concentrations. After this preincubation, 10 *μ*L of 1% starch solution in 20 mM of sodium phosphate buffer, used as substrate, was added to each sample and the reaction mixtures were again incubated at 25°C for 10 min. The reaction was stopped with 20 *μ*L of dinitrosalicylic acid color reagent. The test tubes were then incubated in a boiling water bath for 10 min and cooled at room temperature. The reaction mixture was diluted by adding 300 *μ*L of distilled water and the absorbance was measured at 540 nm. The absorbance of blank samples (in which enzyme solution was added during the boiling) and negative controls (10% methanol-buffer solution in place of extract) were recorded. Acarbose was dissolved in 10% methanol-buffer solution and tested at different concentrations, and it was used as positive control. Analyses were performed in triplicate and the final sample absorbance (*A*_540_ nm) was obtained by subtracting its corresponding sample blank reading [20]. The inhibitory activity was calculated by using the formula and compared to the positive control:

(2) %Inhibition=Abs540  negative  control−Abs540  sampleAbs540  negative  control×100.

The concentration of the extract required to inhibit the activity of the enzyme by 50% (IC_50_) was calculated by regression analysis.

#### 2.7.2. *α*-Glucosidase Inhibitory Activity

The inhibitory activity of *α*-glucosidase enzyme was assessed in 96-well plates, using the procedure previously reported [21]. In each well 40 *μ*L of extract was dissolved in 10% methanol-buffer solution and tested at different concentrations; 130 *μ*L of 10 mM phosphate buffer pH 7.0 and 60 *μ*L of substrate (2.5 mM 4-nitrophenyl *α*-D-glucopyranoside in 10 mM phosphate buffer) were added. The reaction was initiated by the addition of 20 *μ*L of enzyme (0.28 U/mL in 10 mM phosphate buffer) and the plates were incubated at 37°C for 10 min. The absorbance at 405 nm was measured before the addition of the enzyme (*T*_0′_) and after 10 minutes of incubation (*T*_10′_). Acarbose was dissolved in 10% methanol-buffer solution and tested at different concentrations, and it was used as positive control. Negative control absorbance (10% methanol-buffer solution in place of extract) was also recorded. The inhibitory activity was calculated by using the formula

(3) %Inhibition=Abs405  negative  controlT10′−T0′−Abs405  sampleT10′−T0′Abs405  negative  controlT10′−T0′×100.

The concentration of the extract required to inhibit the activity of the enzyme by 50% (IC_50_) was calculated by regression analysis.

### 2.8. Statistical Analysis

Data are expressed as the mean ± standard error (SEM) of three independent experiments. To verify the correlations among used methods, Pearson coefficient was determined and *p* values of 0.05 or less were considered statistically significant. Statistical analyses were performed using GraphPad Prism 5 Software (San Diego, CA, USA).

## 3. Results and Discussion

### 3.1. Total Polyphenols, Tannin Content, and Antioxidant Activity

The total polyphenol content (TPC) and total tannin content (TTC) of olive fruits were analysed (Table 1). Each cultivar showed a different phenolic content; in particular TPC ranged from 147.13 ± 6.94 to 290.21 ± 13.21 mg GAE/g of extract in* Sigoise* and* Coratina*, respectively. TTC, instead, varied from 20.08 ± 3.12 to 86.86 ± 8.74 mg TAE/g of extract in* Sigoise* and* Leccino*, respectively. Between Algerian cultivars we have found both the highest TPC (272.83 ± 9.84 mg GAE/g of extract) and TTC (81.28 ± 10.69 mg TAE/g) in* Chemlal*, while among Italian cultivars the lowest TPC and TTC values were found in* Maiatica* (182.35 ± 7.54 mg GAE/g) and* Ogliarola* extracts (57.51 ± 5.54 mg TAE/g of extract), respectively. No single assay can represent total antioxidant capacity and for this reason 3 different and complementary assays were used to evaluate extract antioxidant activities [17]. Radical-scavenging capacity was determined by DPPH test. Results demonstrated that* Coratina* cultivar (202.62 ± 14.85 mg TE/g) and* Chemlal* cultivar (172.41 ± 11.42 mg TE/g) had the highest radical-scavenging activities (Table 2). The ability of plant extracts to reduce ferric ions was determined by FRAP assay. FRAP test revealed that* Frantoio* cultivar had the highest reducing power (226.44 ± 20.19 mg TE/g), whereas* Sigoise* showed the lowest radical-scavenging (27.40 ± 2.31 mg TE/g) and reducing power (36.34 ± 4.41 mg TE/g) activities.* Coratina*,* Chemlal*, and* Maiatica* also showed a high reducing ability (Table 2). To get a wider overview of the antioxidant potential, the inhibition of lipid peroxidation by BCB test was also carried out. All extracts showed moderate *β*-carotene bleaching inhibition activity; in fact results ranged from 21.95 ± 1.45 to 35.18 ± 2.71% and the highest value was found in* Ogliarola* extract at the final concentration of 0.1 mg/mL.

Pearson coefficient was used to determine the correlation between phenolic compounds and antioxidant activity (Table 3). The highest positive correlation was observed between total phenolic content and DPPH scavenging capacity underlining a strong dependence (*r* = 0.90). This result is in agreement with previous findings [14]. Positive, but lower correlation was obtained between phenolic content and reducing power (*r* = 0.61). Tannin content showed similar Pearson coefficient with radical-scavenging activity (*r* = 0.68) and reducing power (*r* = 0.63). No correlation was observed between lipid peroxidation inhibition (BCB) and phenolic or tannin content. This is probably due to the presence of lipophilic minor compounds acting synergistically enhancing the biological activity. Phenolic compounds including tannins are secondary metabolites known as hydrophilic antioxidants and probably they may show a higher activity in aqueous systems (FRAP and DPPH). In fact it was previously assessed that this kind of behaviour could be due to the different types of antioxidants that are assayed by different methods. TPC gives an indication of the levels of both lipophilic and hydrophilic compounds. BCB in contrast mainly gives an indication of the levels of lipophilic compounds [22].

**Figure 1:**
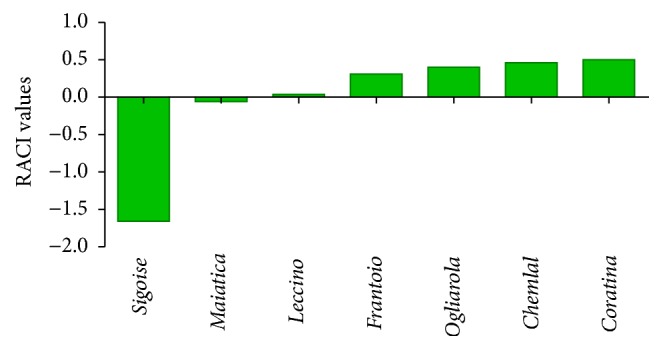
RACI values obtained comparing DPPH, FRAP, BCB, and TPC results. DPPH: 2,2-diphenyl-1-picrylhydrazyl; FRAP: ferric reducing antioxidant power; BCB: *β*-carotene bleaching assay; TPC: total polyphenolic content; RACI: relative antioxidant capacity index.

The integration of antioxidant capacity results derived from different chemical methods allowed calculating the Relative Antioxidant Capacity Index (RACI) among all tested extracts (Figure 1). TPC was also included; in fact it was recently proposed that results obtained by Folin-Ciocalteu procedure could be also interpreted as an alternative way to measure the total reducing capacity of extracts as the reagent reacts with any reducing substance [17]. Data revealed that* Coratina* cultivar had the highest RACI (0.50), followed by* Chemlal* (0.46) and* Ogliarola* cultivars (0.40). According to RACI results,* Sigoise* showed the lowest value (−1.66) between the Algerian cultivars, while* Maiatica* and* Leccino* showed the lowest RACI among Italian cultivars.

### 3.2. HPLC-DAD Analysis

The total phenolic content measured by the Folin-Ciocalteu procedure does not give a full picture of the qualification or quantification of the phenolic constituents in plant matrices [23]. Olive fruit extracts were analysed by a RP-HPLC technique coupled with Diode-Array detector in order to identify and quantify the major phenolic compounds in the selected* Olea europaea* cultivars. Fourteen phenolic compounds were identified by the retention times of the standards. Quantitative data were calculated from their respective calibration curves. Identified compounds can be divided into 4 different classes: phenolic acids (*p*-hydroxybenzoic acid, vanillic acid, caffeic acid, gallic acid, syringic acid,* p*-coumaric acid, ferulic acid, and sinapic acid), flavonoids (luteolin and chrysoeriol), phenolic alcohols (hydroxytyrosol and tyrosol), and secoiridoids (oleuropein and verbascoside). Results of quantification, expressed as mg of single standard/Kg of dried extract, are reported in Table 1. Data showed that amongst phenolic acids the major compound present in the extracts was identified to be gallic acid with an average value of 707.12 mg/Kg and* Sigoise* cultivar was found to be the one containing the highest amount of this phenolic acid (1440.91 ± 20.01 mg/kg) followed by* Frantoio* and* Leccino*, whereas* Chemlal* had the lowest content with only 13.42 ± 2.41 mg/Kg. Luteolin (mean value 831.89 mg/Kg) and oleuropein (mean value 893.77 mg/Kg) were the most abundant compounds amongst flavonoids and secoiridoids, respectively (Table 1).* Leccino* showed the highest content of luteolin (2828.86 ± 107.24 mg/Kg), whereas* Frantoio* showed the highest content of oleuropein (2562.63 ± 46.88 mg/Kg). Hydroxytyrosol was one of the major phenolic compounds in all olive fruit extracts with an average of 2152.81 mg/kg. All cultivars, except* Sigoise* (245.23 ± 25.81 mg/Kg), showed high contents of this compound. Vanillic acid was identified in traces in* Leccino* extract, and* p*-hydroxybenzoic acid was not detectable in* Chemlal* and* Coratina*. In conclusion, gallic acid, hydroxytyrosol, luteolin, oleuropein, and also verbascoside were the most abundant compounds in our extracts. Our results were in accordance with those of Vinha et al. [6] where they found hydroxytyrosol and oleuropein as the two major compounds identified. The concentrations of these two compounds were comparable with ours, but in our case also luteolin was the most abundant flavonoid while they have detected its glycoside derivative as one of the most abundant ones.

Pearson correlation was used to identify the contribution of single compounds to antioxidant activity (Table 3).* p*-Hydroxybenzoic acid and verbascoside were the contributors of reducing power (*r* = 0.93 and *r* = 0.54, resp.), whereas tyrosol seems to be involved in radical-scavenging ability (*r* = 0.61). These results are congruent with previous findings [24, 25].* In vivo* and* in vitro* studies suggest that all these bioactive compounds exhibit a wide range of physiological properties, antiallergenic, anti-inflammatory, anti-microbial, and antioxidant activities [4, 26].

### 3.3. Antidiabetic Activity

The inhibition of *α*-amylase and *α*-glucosidase enzymes in the small intestine is important in the control of type 2 diabetes that is characterized by a rapid increase in blood glucose levels due to hydrolysis of starch by *α*-amylase and the consequent absorption of glucose. The consumption of natural inhibitors from constituents in the diet or as nutraceutical formulation could be an effective therapy for managing postprandial hyperglycemia [27–29].

**Figure 2:**
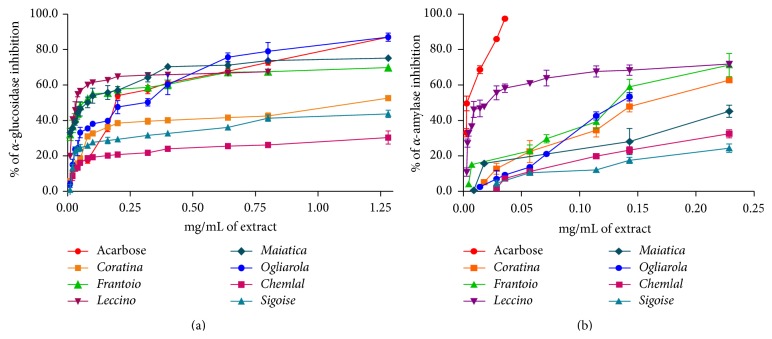
Concentration-dependent inhibition of (a) *α*-glucosidase and (b) *α*-amylase by* Olea europaea* extracts* versus* acarbose.

In our study* Olea europaea* fruit extracts and their most abundant phenolic compounds (hydroxytyrosol, luteolin, oleuropein, tyrosol, and verbascoside) have been evaluated for their ability to inhibit the *α*-glucosidase and the *α*-amylase enzymes. Both acarbose and* Olea europaea* extracts were able to inhibit both enzymes in a concentration-dependent manner as reported in Figure 2. Acarbose was significantly more effective than extracts to inhibit *α*-amylase, whereas comparable results were observed in the *α*-glucosidase inhibition. Results were expressed as IC_50_ (Table 4) and among all extracts,* Leccino* cultivar extract was found to be the best inhibitor of both enzymes, showing the lowest IC_50_ values, 5.31 ± 0.03 *μ*g/mL for *α*-glucosidase, sensibly lower than acarbose (IC_50_ = 340.03 ± 25.12 *μ*g/mL), and 54.70 ± 1.14 *μ*g/mL for *α*-amylase.* Maiatica* extract reported a good *α*-glucosidase inhibition activity (IC_50_ = 297.32 ± 12.41 *μ*g/mL), whereas* Frantoio* and* Ogliarola* cultivars inhibited both enzymes. Algerian cultivars, instead, demonstrated the highest IC_50_ values with both* in vitro* assays.

The results of enzymatic activity of hydroxytyrosol, luteolin, oleuropein, tyrosol, and verbascoside, the most abundant phenolic compounds in investigated* Olea europaea* cultivars, were compared with the extracts and acarbose (Table 4). All single compounds reported IC_50_ values lower than extracts in the *α*-amylase inhibition test, but oleuropein and tyrosol. On the other hand even the most active compounds showed IC_50_ values higher than acarbose (6.75 ± 0.15 *μ*g/mL). Instead, in the *α*-glucosidase inhibition test, hydroxytyrosol, luteolin, and oleuropein reported IC_50_ values lower than both extracts and acarbose. However luteolin compound showed the best *α*-glucosidase and *α*-amylase inhibition activities with IC_50_ values of 14.12 ± 0.85 and 36.09 ± 1.99 *μ*g/mL, respectively. Luteolin was found at the highest amount in the* Leccino* extract and this may explain the marked inhibitory activity present in this cultivar. This result is in agreement with what is previously reported in the literature [30] but not with the Pearson coefficient that we have found among enzymatic inhibition activities and pure compound amounts (data not shown), suggesting the importance of minor compounds in analysed extracts.

The enzymatic inhibition of* Olea europaea* extracts is considerably higher than the enzymatic inhibition reported for some plants used in the treatment of diabetes, such as* Viscum album* (IC_50_ = 11.7 mg/mL),* Glycyrrhiza uralensis* (IC_50_ = 20.1 mg/mL), and* Spergularia rubra* (IC_50_ = 2.55 mg/mL) [21].

The inhibition of *α*-glucosidase and *α*-amylase may be one of the mechanisms involved in the hypoglycemic effect of* Olea europaea* fruits, according to the olive oil results reported in literature [31].

## 4. Conclusions

The extraction procedure together with chromatographic techniques allowed the identification and quantification of the main bioactive compounds in olive fruit extracts. There were no significant qualitative differences among samples, but it is possible to note important quantitative differences.* Chemlal*, between the Algerian cultivars, and* Coratina*, among Italian ones, had the highest content of polyphenols related to a higher radical-scavenging and reducing power activities. The major contributors of their antioxidant activity were hydroxytyrosol, tyrosol,* p*-hydroxybenzoic acid, and verbascoside. Furthermore* Leccino*, among the studied extracts, and luteolin, among the phenolic compounds, were strong inhibitors of *α*-glucosidase and *α*-amylase enzymes. Results of our work confirmed that olive polyphenols play an important role in human health and can significantly contribute to the prevention of diabetes and consequently prevent cardiovascular diseases, especially in the Mediterranean area where olives are normally used as food [8]. Moreover our paper evidenced that* Olea europaea* extracts may have a direct possible application in the pharmaceutical field, due to the presence of bioactive compounds, more abundant in specific cultivars like* Leccino*,* Chemlal*, and* Coratina*.

## Figures and Tables

**Table 1:** Total polyphenolic and tannin content and quantification of identified compounds in *Olea europaea* fruit extracts.

	Chemlal	Coratina	Frantoio	Leccino	Maiatica	Ogliarola	Sigoise
TPC^*∗*^	272.83 ± 9.84	290.21 ± 13.21	223.81 ± 16.18	224.92 ± 9.26	182.35 ± 7.54	226.89 ± 6.63	147.13 ± 6.94
TTC^*∗∗*^	81.28 ± 10.69	52.92 ± 4.32	63.95 ± 5.36	86.86 ± 8.74	66.27 ± 6.45	57.51 ± 5.54	20.08 ± 3.12
mg of gallic acid/Kg of dried extract	13.42 ± 2.41	191.63 ± 12.01	1349.75 ± 29.31	1097.51 ± 11.52	528.24 ± 33.22	328.35 ± 16.21	1440.91 ± 20.01
*p-*Hydroxybenzoic^*∗∗∗*^	nd	nd	309.36 ± 44.12	66.43 ± 19.74	308.87 ± 20.34	116.42 ± 13.11	3.22 ± 0.87
Vanillic acid^*∗∗∗*^	34.84 ± 7.74	134.66 ± 10.41	203.46 ± 9.14	nd	493.94 ± 14.77	37.53 ± 14.75	200.93 ± 6.41
Caffeic acid^*∗∗∗*^	8.72 ± 0.74	80.65 ± 2.93	142.17 ± 4.47	129.32 ± 1.42	96.46 ± 8.36	83.42 ± 5.74	29.64 ± 0.77
Syringic acid^*∗∗∗*^	6.64 ± 0.62	32.84 ± 1.93	81.65 ± 0.33	63.24 ± 0.89	120.68 ± 1.47	39.12 ± 0.15	13.64 ± 1.81
*p-*Coumaric acid^*∗∗∗*^	17.65 ± 1.61	6.57 ± 0.28	35.74 ± 0.64	21.95 ± 0.84	19.33 ± 0.55	28.74 ± 2.08	66.37 ± 1.11
Ferulic acid^*∗∗∗*^	103.09 ± 1.97	37.78 ± 1.56	25.22 ± 2.27	31.75 ± 1.84	156.54 ± 2.91	31.36 ± 1.76	63.38 ± 0.82
Sinapic acid^*∗∗∗*^	23.46 ± 0.33	30.64 ± 4.16	25.95 ± 2.58	24.22 ± 0.12	44.67 ± 3.47	31.85 ± 0.65	26.34 ± 0.97
Tyrosol^*∗∗∗*^	100.21 ± 1.03	134.75 ± 18.87	200.84 ± 75.12	194.13 ± 27.24	17.96 ± 7.39	115.74 ± 14.91	34.34 ± 3.62
Hydroxytyrosol^*∗∗∗*^	2024.63 ± 26.01	1927.57 ± 104.37	2338.45 ± 82.11	1876.23 ± 123.01	3683.44 ± 364.27	2974.14 ± 23.72	245.23 ± 25.81
Verbascoside^*∗∗∗*^	21.37 ± 0.64	319.78 ± 7.51	693.77 ± 13.66	643.09 ± 18.47	718.68 ± 9.74	335.34 ± 4.16	52.72 ± 0.95
Oleuropein^*∗∗∗*^	109.86 ± 3.68	126.92 ± 13.84	2562.63 ± 46.88	1074.28 ± 47.85	1361.47 ± 71.71	804.56 ± 52.43	216.70 ± 12.34
Luteolin^*∗∗∗*^	201.70 ± 1.55	221.74 ± 6.48	585.64 ± 44.72	2828.86 ± 107.24	513.24 ± 77.64	1362.51 ± 35.67	109.54 ± 1.22
Chrysoeriol^*∗∗∗*^	21.73 ± 0.71	11.68 ± 0.56	135.57 ± 4.13	303.14 ± 7.21	549.25 ± 14.94	158.74 ± 3.87	36.37 ± 0.56

TPC: total polyphenolic content; TTC: total tannin content; values are the mean of three determinations ± standard deviation (*p* < 0.05). ^*∗*^Milligrams of gallic acid equivalents per g of extract; ^*∗∗*^milligrams of tannic acid equivalents per g of extract; ^*∗∗∗*^mg of compound per Kg of extract.

**Table 2:** Antioxidant activity of investigated *Olea europaea* fruit extracts.

Cultivar	DPPH^*∗*^	FRAP^*∗*^	BCB^*∗∗*^
*Chemlal*	172.41 ± 11.42^B^	191.48 ± 8.96^A^	23.44 ± 1.78^B^
*Coratina*	202.62 ± 14.85^A^	200.29 ± 13.25^A^	25.16 ± 2.29^B^
*Frantoio*	163.77 ± 10.32^BC^	226.44 ± 20.19^A^	26.02 ± 2.11^B^
*Leccino*	154.34 ± 16.17^C^	140.07 ± 11.68	21.95 ± 1.45^B^
*Maiatica*	126.94 ± 5.14^D^	210.09 ± 24.91^A^	25.00 ± 2.02^B^
*Ogliarola*	155.96 ± 10.36^C^	149.05 ± 10.25^B^	35.18 ± 2.71^A^
*Sigoise*	27.40 ± 2.31^D^	36.34 ± 4.41^C^	22.09 ± 1.88^B^

DPPH: 2,2-diphenyl-1-picrylhydrazyl; FRAP: Ferric Reducing Antioxidant Power; BCB: *β*-carotene bleaching assay; values are the mean of three determinations. ^*∗*^Milligrams of Trolox equivalents per g of extract; ^*∗∗*^antioxidant activity at [0.1 mg/mL]. Superscripts represent statistical differences between cultivars at *p* < 0.05 using ANOVA with Scheffe post hoc analysis.

**Table 3:** Pearson correlation coefficient calculated among measured antioxidant activities and quantified chemical compounds.

	DPPH	BCB	FRAP
TPC	0.90	0.14	0.61
TTC	0.68	−0.04	0.63
Gallic acid	−0.60	−0.35	−0.46
*p-*Hydroxybenzoic acid	0.54	0.15	0.93
Vanillic acid	−0.29	−0.33	0.16
Caffeic acid	0.36	0.15	0.42
Syringic acid	0.13	0.04	0.50
*p-*Coumaric acid	−0.89	−0.13	−0.77
Ferulic acid	−0.22	−0.25	0.17
Sinapic acid	−0.02	0.28	0.30
Tyrosol	0.61	0.06	0.35
Hydroxytyrosol	0.54	0.52	0.74
Verbascoside	0.30	0.06	0.52
Oleuropein	0.14	0.14	0.46
Luteolin	0.19	0.08	−0.09
Chrysoeriol	−0.04	0.05	0.26

DPPH: 2,2-diphenyl-1-picrylhydrazyl; FRAP: Ferric Reducing Antioxidant Power; BCB: *β*-carotene bleaching assay; TPC: total polyphenolic content; TTC: total tannin content.

**Table 4:** *α*-Glucosidase and *α*-amylase inhibition activities of *Olea europaea* fruit extracts, standard compounds *versus* acarbose (positive control).

Sample	IC_50_ *µ*g/mL	
	*α*-Glucosidase	*α*-Amylase
*Chemlal*	2531.06 ± 238.15^A^	337.30 ± 5.25^A^
*Coratina*	1119.30 ± 25.59^C^	190.40 ± 8.70^C^
*Frantoio*	360.43 ± 15.53^EF^	144.20 ± 5.94
*Leccino*	5.31 ± 0.03^I^	54.70 ± 1.14^CD^
*Maiatica*	297.32 ± 12.41^F^	254.59 ± 1.14^F^
*Ogliarola*	398.43 ± 15.63^E^	154.26 ± 4.28^B^
*Sigoise*	1473.00 ± 16.04^B^	352.28 ± 29.52^CD^
Hydroxytyrosol	14.85 ± 0.91^H^	81.63 ± 7.17^E^
Luteolin	14.12 ± 0.85^H^	36.09 ± 1.99^G^
Oleuropein	177.14 ± 11.53^FG^	181.08 ± 12.75^C^
Tyrosol	391.82 ± 23.15^E^	185.71 ± 18.00^C^
Verbascoside	948.41 ± 30.09^D^	121.33 ± 10.21^D^
Acarbose	340.03 ± 25.12^F^	6.75 ± 0.15^H^

Results are expressed as mean value of triplicate ± standard deviation. Superscripts represent statistical differences between cultivars at *p* < 0.05 using ANOVA with Scheffe post hoc analysis.
